# The preferences of Hungarian judges with regard to forensic psychiatry and forensic psychology experts

**DOI:** 10.1192/j.eurpsy.2024.1207

**Published:** 2024-08-27

**Authors:** B. Baran, F. Á. Szabó, A. Lisincki, L. Vida, P. Czobor, É. Jekkel, B. Szuromi

**Affiliations:** ^1^Psychiatry and Psychotherapy, Semmelweis University; ^2^National Institute of Mental Health, Neurology, Neurosurgery -Nyírő Gyula Center, Budapest, Hungary

## Abstract

**Introduction:**

Although Hungarian forensic psychiatry has a historical legacy dating back to the 1890s, for the past few years there has been a dramatically increasing shortage of forensic psychiatry experts in Hungary, which affects both health care practices and the judiciary.

**Objectives:**

In order to join the international academic unity of forensic psychiatry including research, education and treatment besides expert witnessing, our workgroup aims to facilitate the development of high-level quality standards in modern forensic psychiatry in Hungary. Based on our pilot study on this topic, in the current nationwide study we attempted to delineate the preferences of Hungarian judges regarding the role of forensic psychiatry and forensic psychology experts in both criminal and civil legal proceedings.

**Methods:**

With the help of the National Office of the Courts, Hungarian judges were asked to complete a questionnaire that - besides personal characteristics – comprised specific questions in several areas including; hearing the experts; their preferences when assigning experts; the value of the expert’s oral statement in court; the extent of their reliance on the psychiatric or legal knowledge of the experts; and the ways judges assess their own psychiatric and psychological knowledge. They were also asked to disclose their opinion about the attributes of optimally applicable expert opinions. Respondents provided their answers as rating on a 10-point Likert scale; or as percentage estimates. Besides descriptive statistics, we investigated the difference between the two groups of judges using Chi-square statistics and ANOVA with respect to the association between the answers and the main personal characteristics of judges.

**Results:**

The dataset contains >400 completed questionnaires, returned from all over the country, and the analyses are ongoing. Preliminary results are available for a sample of 125 respondents: 53 criminal court and 72 civil court judges, with 64 of them having a maximum of 14 years and 61 having more than 14 years of work experience. Rating the characteristics of assigned experts, we found a significant association between being a criminal court judge and assigning an expert who is considered as an “acknowledged authority” by peers (p=0.002). Finding it crucial what the assessed people report on the legal case itself was significantly associated with civil court judges (p=0.026).

**Conclusions:**

In the absence of any available nationwide information in Hungary, our study is expected to provide much-needed and fundamental information to the current practice of forensic psychiatry in the country.

**Disclosure of Interest:**

None Declared

